# Italian law on medically assisted reproduction: do women’s autonomy and health matter?

**DOI:** 10.1186/s12905-016-0324-4

**Published:** 2016-07-23

**Authors:** Irene Riezzo, Margherita Neri, Stefania Bello, Cristoforo Pomara, Emanuela Turillazzi

**Affiliations:** Institute of Legal Medicine, Department of Clinical and Experimental Medicine, University of Foggia, Ospedale Colonnello D’Avanzo, Via degli Aviatori, 1, 71100 Foggia, Italy

**Keywords:** Italian law, Medically assisted reproduction, Reproductive choices, Autonomy, Discrimination

## Abstract

**Background:**

In Italy in 2004, a very restrictive law was passed on medically assisted reproduction (MAR) (Law 40/2004) that placed Italy at the most conservative end of the European spectrum. The law was widely criticized and many couples seeking MAR brought their cases before the Italian Civil Courts with regard to pre-implantation genetic diagnosis (PGD), donor insemination and the issue of consent. Ten years on, having suffered the blows of the Italian Constitutional Court, little remains of law 40/2004.

**Discussion:**

In 2009, the Constitutional Court declared the maximum limit of the number of embryos to be produced and transferred for each cycle (i.e. three), as stated in the original version of the law, to be constitutionally illegitimate. In 2014, the same Court declared as unconstitutional the ban on donor insemination, thus opening the way to heterologous assisted reproduction. Heterologous MAR is therefore perfectly legitimate in Italy. Finally, in 2015 a further ruling by the Constitutional Court granted the right to access MAR to couples who are fertile but carriers of genetic diseases. However, there is still much room for criticism. Many couples and groups are still, in fact, excluded from MAR. Same-sex couples, single women and those of advanced reproductive age are, at the present time, discriminated against in that Italian law denies these subjects access to MAR.

**Summary:**

The history of Law 40/2004 has been a particularly troubled one. Numerous rulings have, over the years, dismantled much of a law constructed in violation of the rights and autonomy of women and couples. However, a number of troubling issues still exist from what is left of the law and the debate is still open at national and transnational level regarding some of the contradictions and gaps in the law highlighted in this article. Only by abolishing the final prohibitions and adopting more liberal views on these controversial yet crucial issues will Law 40/2004 become what it should have been from the start, *i.e.* a law which outlines the ‘rules of use’ of MAR and not, as it has been until now, a law of bans which sets limits to the freedom to reproduce.

## Background

In 2004, the Italian parliament promulgated a law (law 40/2004) on medically assisted reproduction (MAR). Over the years, all previous attempts to introduce legislation in this field had failed because of bitter contention, in both the Italian cultural and juridical fields, between those who consider it necessary to have rigorous and detailed legislative control and those who regard only minimal legal intervention necessary in relation to the access and use of MAR [1].

It had become apparent over the years that some sort of regulation of MAR was necessary in Italy. Firstly, there was a highly competitive commercial environment with little regulation or supervision prior to the approval of the law [2]. Moreover, also prior to the law, Italian judges were frequently required to rule on cases of disownment of paternity subsequent to MAR [3], posthumous insemination [4], and surrogate motherhood [5]. Since there was no specific law to regulate these sensitive issues, juridical outcomes often differed.

The Italian legislature preferred to lay down a very restrictive law [6] that placed Italy at the most conservative end of the spectrum in Europe [2].

The law begins with the statement that recourse to MAR is allowed only in order to help solve reproductive problems arising as a result of sterility or infertility, so as to guarantee the rights of all people involved, including those of the conceived child. Since protection of the child born from MAR was the chief concern, the law prohibited paternal disownment. Furthermore, acknowledgement of the child born from MAR was made compulsory for the mother. The law provided the same rights to children born under MAR techniques as to children conceived naturally [1]. It set up a national register of authorized centres for MAR techniques and established strict technical and safety requisites for the centres themselves. The register collects the data from most of the fertility centres, and monitors the safety and the effectiveness of MAR procedures. A constant quality check on both the operators’ professionalism and the adequacy of the equipment and applied technologies of the authorized centres was guaranteed. This is a valuable aspect of the law since it undoubtedly provides a guarantee for women undergoing MAR.

Originally, the law contained several bans, as well as subjective requirements for couples who could access MAR. Couples had to be adults of different genders, of potentially fertile age and affected by sterility or infertility. They had to be married or living together and both living. Gamete donation and surrogate motherhood were banned. The law also established that a maximum of three embryos could be produced and used in one ‘sole and simultaneous implant’, forbidding, at the same time, the cryopreservation of embryos, except when implantation was temporally impossible due to the mother’s transitory health problems. As a result, PGD was banned [1].

## Discussion

### Overview and facts

The law was widely criticized [7–12] and Italian Courts heard more than 30 challenges to various aspects of the law itself [13]. In particular, many Italian couples applied the Civil Courts to be granted access to PGD and donor insemination [13].

Strict interpretations of both the law and the Ministry of Health’s subsequent guidelines led to infertile couples affected by genetic diseases being denied the right to resort to PGD. Over the years, rulings from many Italian Civil Courts (Court of Cagliari, Court of Florence, 2007) [14] have recognized the right of infertile couples affected by genetic diseases to access PGD. Through PGD it is possible to define the genetic composition of embryos prior to embryo transfer. Chromosomal abnormalities, X-linked diseases or single gene disorders are the well-known medical indications for PGD [15]. Scientific debate widened to include other controversial uses such as inherited cancer predispositions [16], human leukocyte antigens (HLA) typing used to select (an) embryo(s) for intrauterine transfer with a view to giving birth to an unaffected HLA matching sibling [17], sex-selection for non-medical reasons [18], all of which sparked heated ethical discussion [19, 20].

In Europe at the time, national regulations differed across the board. In some countries, such as Germany and Switzerland, PGD was prohibited by law, whereas in France, the United Kingdom, Spain, Denmark, and Belgium it was permitted. In countries such as Finland and Portugal, PGD was practised without government regulations [15].

Within this international scientific and legislative background, the Italian Constitutional Court in 2009 finally declared that the maximum limit of three embryos was constitutionally illegitimate, as was the consequent obligation to implant all three embryos produced, thus opening the way for infertile couples to resort to PGD [21].

The consequences were not slow in coming, and resulted in deep changes on the Italian reproduction scene [22, 23]. The official figures for 2012 (Ministry of Health) showed a change in the application of MAR procedures compared to the period before the 2009 Constitutional Court ruling: an increase in cycles by embryo thawing techniques and a decrease in fresh technique cycles, compared to a steady increase in these cycles in previous years. Thawing and freezing procedures increased for embryos and decreased for oocytes. Analysis shows that following legalization, the number of referrals asking for PGD significantly increased [24, 25]. These results strongly indicate that Italian women and couples contemplating a future pregnancy would consider the use of PGD.

PGD was a particularly troubled issue. Until 2015, couples who were fertile but carriers of genetic disease were discriminated against, in that the Italian law denied these couples access to MAR and PGD, which could only be carried out as part of MAR procedures following the 2009 ruling of the Constitutional Court. Couples who were fertile but carriers of genetic disease brought the question of their right to obtain PGD to the attention of Italian Civil Courts (Court of Salerno, 2010). Judges allowed these fertile couples to resort to assisted reproduction, and above all, to PGD. In 2012 the European Court of Human Rights (ECtHR) (28 August 2012) affirmed that the prohibition to access MAR for fertile couples violated article 8 of the European Convention on Human Rights (ECHR) and condemned Italy [26, 27]. Recently, the Court of Rome has questioned the constitutional legitimacy of forbidding access to fertile couples who are carriers of transmissible genetic disease which, in fact, prevents aware and responsible procreation. Civil judges underlined the fact that the limit of access to MAR for fertile couples violates the right of such couples to health and autonomy as well as the right to procreate. In such cases, in an attempt to fulfil a legitimate desire to have a child unaffected by a serious genetically transmissible disease, the couple, and particularly the woman, must embark on a natural pregnancy knowing that she might have to abort, with increased risk for her physical and mental health. On 5 June 2015, the Constitutional Court ruled that also fertile couples who are carriers of transmissible genetic disease have the right to access MAR and PGD.

### Donor insemination

In 2010 and 2011, following the requests of infertile couples, the Civil Courts of Milan, Florence and Catania questioned the prohibition of gamete donation imposed by law 40/2004 and referred this issue to the Constitutional Court [13]. In April 2014, the Court (n. 162/2014) condemned as unconstitutional the ban on heterologous fertilization. Until this judgment, the restrictions imposed by the law had driven many infertile Italian couples to choose to go abroad so as to have wider access to assisted procreative treatments [28–30]. A 2010 study showed that of the six countries studied, Italy was in first place for cross-border reproduction care: Italian patients numbered 31.8 % of the total [31]. This figure has remained constant since the first study by the national Observatory on Procreative Tourism, testifying to the fact that the demand for infertility treatments in Italy has not received an adequate response [32]. In 2011, at least 4,000 Italian couples travelled abroad: half of these found it necessary to leave Italy because they sought donor insemination; the other half left for no apparent reason to have treatment which was also available in Italy. This trend has remained constant since the law came into force when, in a single year, there was an almost 200 % increase in the number of Italian couples who went abroad for reproductive reasons. This phenomenon constitutes a reproductive ‘exile’ [33] in that Italian infertile couples feel barred from accessing MAR in their home country.

In the rulings mentioned above, Italian judges raised doubts regarding the constitutional legitimacy of the ban on donor insemination imposed by the law. All infertile couples should have an equal opportunity to resort to the most effective MAR procedures so as to resolve their inability to have children. Because of this ban, couples affected by infertility which could not be treated unless by gamete donation were denied the right to satisfy their desire for a child and make their own procreative choices. The ban on sterile couples resorting to gamete donation was, therefore, a violation of personal liberty, autonomy and the right to comprehensive healthcare for women that must include birth control, abortion and sexual and reproductive health. Furthermore, this ban represented a great disparity in health care, creating a gap between couples who could, legitimately, fulfil their desire for a child and achieve full reproductive health through approved assisted reproduction and the more numerous group whose rights were denied because they could not resort to gamete donation. This was also a violation of the Italian Constitution (art. 2) which ensures equal rights to all citizens, equal medical care (art.32), and also of the National Health System law which guarantees comprehensive healthcare for all citizens without discrimination. On the contrary, blatant discrimination exists, on an economic basis, between couples with fertility problems: only those with economic means to seek treatment abroad can fulfil their desire to have a child.

The poignancy of the quests of infertile Italian couples for heterologous MAR is further demonstrated by the fact that immediately following the Constitutional Court judgment on heterologous MAR, there were approximately 3,500 queries from couples to access donor insemination at the Italian CeCOS centres (centres for the study and preservation of human oocytes and sperm) [34], once again reflecting the dissatisfaction of infertile women and couples regarding the deficiencies in their home country.

The response of the constitutional judges was unequivocal. A couple’s decision to become parents and to form a family is an expression of their fundamental freedom to self-determination which concerns both personal and family matters. The decision on whether or not to have a child affects the most intimate and untouchable part of the individual. As such, this decision must be incoercible, as long as it does not endanger other constitutional values, and even if it involves resorting to MAR procedures with donors.

In the judgment of April 2014, the Constitutional Court ruled that gamete donation techniques were immediately applicable within the regulatory framework currently in force. There is no danger of a legislative void since all the unchanged/unrepealed parts of law 40/2004 remain in force. Heterologous MAR is therefore perfectly legitimate ‘even if only in different-sex couples, married or cohabiting, and only in potential childbearing age women’. However, many factors may still hinder women’s and couples’ access to heterologous MAR.

### Recommended changes

There are several controversial aspects of Law 40/2004, some of which derive from the original form of the law, including criteria for accessing therapy, and withdrawal of consent to MAR procedures.

The Italian law states that access to MAR should be limited to adults of different genders, of fertile age, with medically-certified sterility or infertility. They should be married or living together, and both living. In 2007, following a Ministerial Decree, a sexually transmissible disease in the man was deemed to be a condition of sterility such as to guarantee access to MAR for these couples.

However, many couples and groups are still excluded from MAR in Italy. Same-sex couples, single women, post-mortem insemination, surrogacy and gestational carriers are still banned. These are challenging and unresolved topics non only in Italy [35] but everywhere [36, 37].

Whether this discriminates against single women or homosexual couples, or whether it is an affirmation of the rights of children born from MAR to grow up in a traditional family with both male and female role models for their healthy psychosocial development, is still a matter for debate. There are studies which demonstrate that raising children in family units with homosexual parents does not have a significant negative impact on cognitive development and function, emotional adjustment, gender identity or behaviour when compared with children of heterosexual couples or single mothers [38, 39]. However, there is some debate about the risks for the child. This mainly concerns the psychosocial risks of growing up in a non-nuclear familial environment. Critics fear negative repercussions for the wellbeing of the child (and, later, of the adult) [40]. However, results from empirical studies so far are to a large extent reassuring [41]. In the recent position statement of ESHRE’s Task Force on Ethics and Law, it is reported that ‘assisted reproduction in non-standard situations is morally sound in many cases. There is no good reason to *a priori* dismiss access in these situations – such categorical dismissal would imply discrimination’ [40].

In its present form, though with changes introduced following the numerous decisions by Italian judges, law 40/2004 still reinforces and protects a very narrow concept of the ‘appropriate’ family unit as being one composed of children with heterosexual parents who are married or in a stable cohabiting relationship. This is in opposition to the rights and autonomy of women and couples in relationships to reproductive freedom, stressed as the basic right of all couples and individuals to make free and accountable decisions. And yet in Italy, despite the impact of prevalent Catholic thought, and albeit more slowly than in other countries, profound changes are occurring in the traditional family, with a higher number of single-parent families, single person households, childless couples, and same-sex couples [42]. A recent Italian study suggested that, despite the societal pressures that Italian same-sex couples have to face, these relationships appear resilient, and fare well both overall and in the specific domains of functioning compared to heterosexual couples both in Italy and the United States [43].

There is a lack of consensus among Italian healthcare professionals regarding the bans imposed by the law. For example, in a recent cross-sectional study involving 224 healthcare professionals working with assisted reproduction in Brazil, Italy, Germany and Greece, it was reported that even in Germany and Italy, where insemination is illegal for single women, almost a quarter of the professionals (24.4 %) claimed that they would agree to perform the procedure [44]. This highlights once again the gap that exists not only between Law 40/2004 and Italian couples, but also between the law and Italian healthcare professionals. Thus, the law does not seem to reflect the new social needs and the ongoing cultural changes that are now present in Italy.

A further point of great concern is the withdrawal of consent to MAR procedures. The law underlines the need for informed consent, which must be expressed in writing by both members of the couple. This can be revoked by either person until the time the oocyte is inseminated. From this moment on, the physician may decide not to proceed solely on medical grounds. The law states (art.6) that the woman cannot withdraw consent to the technique from the time of fertilization. Respect for autonomy is a central principle in Italian healthcare, and it implies that patients should not be coerced into medical treatments. The idea that the patient’s autonomy is something valuable that has to be protected emanates from the Italian Constitution (articles 2,3, 13, and 32). Article 6 of Law 40 is a striking example of how this law infringes upon women’s autonomy and appears to be the result of assigning a higher value to the protection of embryos than to the interests and rights (including autonomy) of infertile women. As consent withdrawal is still not permitted by the law, the protection of women's right to autonomy and health requires careful consideration. We argue that respect for women’s personal decisions constitutes a reason to oppose this article, whose dubious legitimacy has been highlighted in recent years by the Civil Court of Florence (2008). However, at the time of writing there has been no definitive ruling by the Italian Constitutional Court on this matter. Finally, women who are not allowed to withdraw their consent to the technique from the time of fertilization, may paradoxically, once pregnancy has begun, make the decision to terminate the pregnancy, under the Italian law (194/1978) on voluntary termination of pregnancy. However, women's experiences of abortion are complex; confronting an unwanted pregnancy is not easy and deciding to terminate it is anything but straightforward. Abortion is a stressful experience [45], and the risks of both surgical and medical abortion for women’s health are well known [46]. Thus it seems that prohibiting the withdrawal of consent is still deeply problematic for the health and autonomy of Italian women.

Other issues have arisen as a result of the recent lifting of the ban on heterologous MAR. Following the decision of the Constitutional Court, and in the silence of the national legislators who have not yet seen fit to intervene, Italian regions have drawn up a document setting out operational guidelines and homogeneous clinical indications to facilitate the immediate granting of the right to heterologous MAR throughout the country.

The following are the key points: adequate technical provisions for the selection of donors and the safety of the donation in compliance with the EU Tissue and Cells Directive (EUTCD) and the supplementary technical directives 2006/17/EC and 2008/86/EC; the establishment of a national register for tracing donor/child; the unpaid and voluntary nature of gamete donation; the introduction of a maximum limit of births from the same donor; the introduction of a minimum and maximum age for donors; the anonymity of gamete donors.

The debate on open or anonymous gamete donation continues unabated [47, 48], While anonymous gamete donation has long been the preferred practice, a new focus on the rights and interests of donor-conceived children has led a number of countries to shift towards an open-identity system. Recently, the Ethics Committee of the American Society for Reproductive Medicine, while acknowledging that these are very personal choices and that the people involved can have very different opinions [49], came out in favour of disclosure to the child of donor conception and, if available, any characteristics of the donor that may serve the best interests of offspring [50]. Some authors have analysed this issue in terms of asymmetries of harm and benefit for both children and donors.

The open system recognizes the child’s right to know his/her biological lineage in that it is fundamental to the donor offspring’s wellbeing and understanding of his/her identity, as well as to choices made later in life [51–53]. Furthermore, disclosure is the basis for open and honest communication with children in order to avoid oppressive family secrets which may have possible negative repercussions for family relationships [54–56]. There are, however, those who would argue that in case of gamete donation there are compelling reasons for not telling the child. A reason that is often given is that it is not in the best interests of the child to know [57]. If facts about a child’s conception spread throughout the family and school environment, this could lead to isolation and stigmatization [58]. Finally, some authors affirm that parents have a right to privacy which would be violated were the details of conception to be released [59]. An evolving debate is now underway regarding the other parties involved, and the possible right of the donor to information about the offspring conceived by their donation is emerging [60]. The exchange of medical information between donors and donor-conceived individuals could ensure the donor’s health since the latter’s access to medical information regarding their offspring (for example in the case of genetic disease) may obviously be relevant for clinical reasons [61]. It has been argued that information regarding offspring would allow donors to feel positive about their donation, thus improving the psychological and social wellbeing of donors themselves [60]. This does not automatically entail parental disclosure [60]; however, identifying information could allow the opportunity for donors to have contact with their offspring [60, 62]. It could be beneficial for donors willing to know their offspring, although this could open risky scenarios in which potential dangers for offspring have to be taken into account if social parents have not informed the child about his/her conception, and if offspring and parents are not willing to meet donors [60].

In spite of this complex debate and of the international trends, Italy is moving towards a system of anonymous gamete donation with a maximum limit of 10 children born from each donor.

In conclusion, Law 40/2004 has been profoundly changed over the years following the judgments of the Italian Constitutional Court that have guaranteed more people the right to access MAR, PGD and donor insemination. However, since issues such as the ban of withdrawal of consent to the procedure until the time the oocyte is inseminated, and the further liberalization of the actual criteria to access MAR (single women, lesbian and gay couples) still remain unresolved, the decisions of the Constitutional Court are unlikely to end the debate on MAR.

## Conclusions

The story of Law 40/2004 has been a particularly troubled one [63]. Numerous rulings have, over the years, dismantled much of a law constructed in violation of women’s and couples’ rights and autonomy. There have been many steps forward; yet the Italian law still does not guarantee that the right to raise a family, which is protected by the Italian Constitution, is supported by the State’s duty to offer the best treatments available for all women and couples seeking a child. A number of troubling issues still arise from what is left of the law and the debate is still open at national and transnational level on some of the gaps in the law highlighted by this article. The issues surrounding assisted reproduction are the common outcome of advanced nations’ technological and cultural development, and so need to be studied from a transnational perspective, keeping in mind the global landscape of cross-border reproductive care [64]. Within Europe at least 24,000-30,000 cycles are performed annually on at least 11,000-14,000 foreign patients [31].
